# Effect of compressibility and non-uniformity in flow on the scattering pattern of acoustic cloak

**DOI:** 10.1038/s41598-017-02143-y

**Published:** 2017-05-18

**Authors:** Hyeonbin Ryoo, Wonju Jeon

**Affiliations:** Department of Mechanical Engineering, Korea Advanced Institute of Science and Technology, Daejeon, 34141 Korea

## Abstract

During the last decade, most of acoustic cloak research has been done within a theoretical framework in which the medium is at rest. However, such an acoustic cloak cannot preserve its unique properties or functions to make an object acoustically invisible in the presence of flow. In this study, we propose a theoretical framework to accurately investigate the effect of compressibility and non-uniformity in flow on the scattering pattern of acoustic cloak. In the formulation, the wave operator is coupled with the non-uniform velocity vector, and the equivalent source terms due to mean flow are divided into the compressibility effect and the non-uniformity effect with their own physical meanings. Numerical simulation shows the difference in far-field directivity between previous and present formulations. The polarity of the equivale﻿nt s﻿ources ﻿in the present formulation shows hexapole and skewed quadrupole patterns for non-uniformity and compressibility effects, respectively, and their magnitudes increase with power laws of ﻿Mach ﻿number as the Mach number increas﻿es. As an application, we make use of the present formulation for predicting the acoustic scattering from newly designed convective cloaks. The simulation results show better performance compared to the existing convective cloak.

## Introduction

During the past 10 years, acoustic cloak has attracted considerable interests because of its unique property to make an object acoustically invisible^1–9^. This unique property is not only academically fascinating in that we can manipulate the acoustic wave mathematically^1–5^, but also practically useful in that it has potential applications in practical engineering problems such as submarines^7^ or aircrafts^6,9^.

Since the theory of acoustic cloak^1–5^ was developed by its analogy with the one of optical cloak^10,11^, it is worth mentioning the previous works in both transformation optics and transformation acoustics. In 2006, electromagnetic cloaks were presented independently by Pendry *et al*.^10^ and Leonhardt^11^ based on diffeomorphism and coordinate transform. In the same year, Milton *et al*.^12^ published a paper on mechanical cloak based on the analogy between electromagnetism and elastodynamics. In 2007, Cummer and Schurig^1^ suggested a two-dimensional acoustic cloak using the equivalence between acoustic equations and single polarization Maxwell equations. Since then, there were many papers on acoustic cloak research, but most of them have been done within a theoretical framework in which the medium is at rest^1–5^. However, unlike the optical or electromagnetic cloaks, the presence of medium convection is an important and unique feature in acoustic cloaks.

Acoustic cloak in convected media has been rarely studied, but attempted by only a few researchers. As reported in the previous works^6–9^, the conventional acoustic cloak in convective media loses its cloaking properties because of a coupled effect of background flow and acoustic wave. Garcia-Meca *et al*.^6^ suggested an analogue transformation approach to acoustic cloak. They derived a transformation formalism that allows involving *uniform* background flow around a carpet cloak. Huang *et al*.^7^ introduced an analytical framework to design an acoustic cloak in the presence of low subsonic flow. They studied two-dimensional acoustic cloaking shell within *incompressible* flow, and derived the equivalent sources by establishing a convective wave equation whose spatial differential operator is coupled with the *uniform* velocity. Then, the velocity difference from non-uniform velocity was included in a source term. However, since more of the real fluid effects are included in differential operator of convective wave equation rather than in source term^13^–^15^, the effect of *non-uniform* flow needs to be theoretically revisited. Therefore, in this study, we propose a theoretical framework to investigate the scattering pattern of an acoustic cloak encountering monochromatic plane waves within *compressible non-uniform* flow. We make use of the proposed theoretical framework for predicting the acoustic scattering from newly designed cloaks as an example of practical applications.

## Results

### Theoretical framework

In this section, we introduce a theoretical framework to investigate the scattering pattern of plane wave impinging on an acoustic cloak in moving media. Before introducing the theoretical framework, we briefly review the existing frameworks of acoustic cloak research during the past 10 years^1–9^.

**Figure 1:**
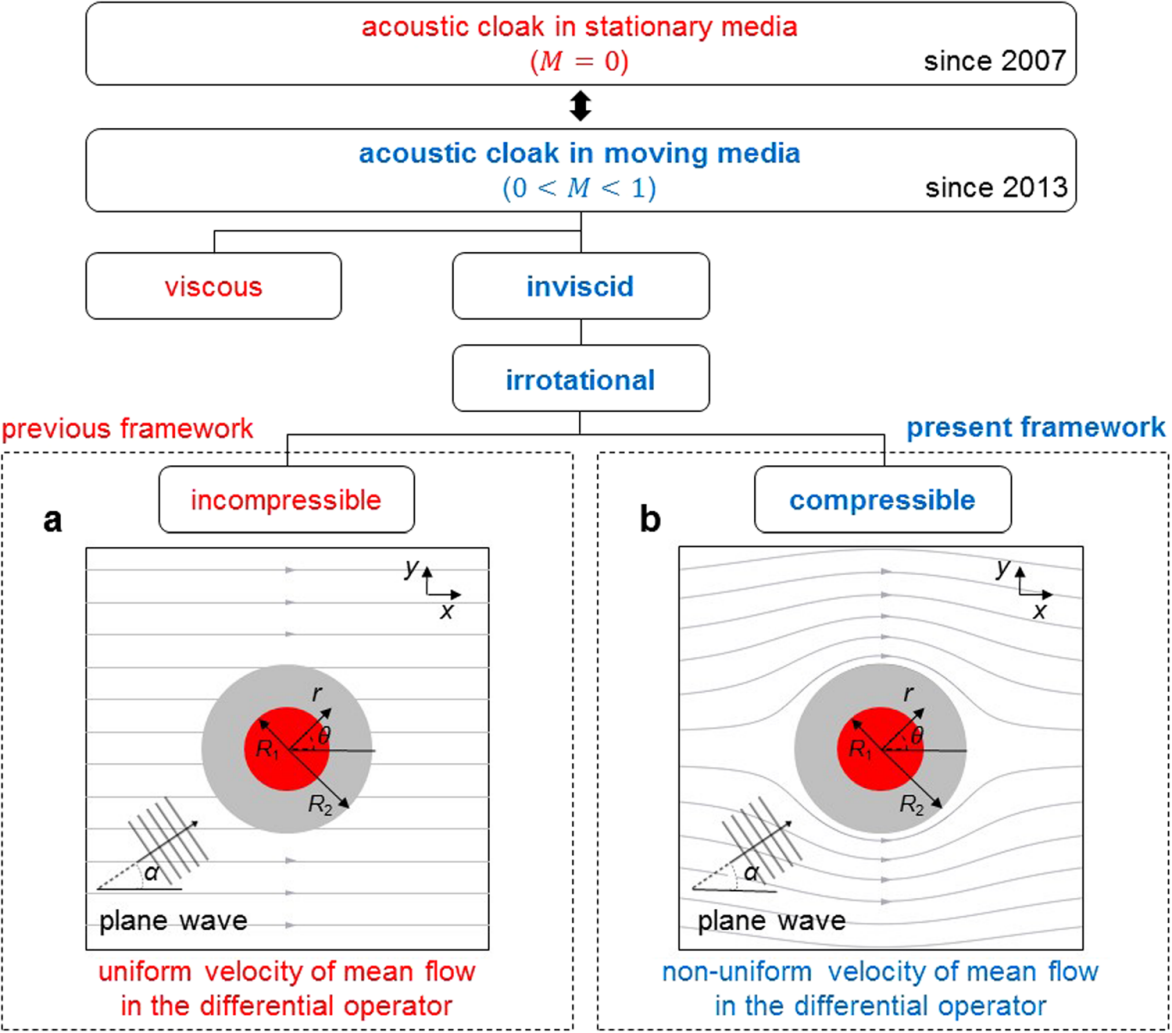
Schematic of acoustic cloak research in stationary and moving media. Early works on acoustic cloak have been done in the absence of flow for a decade. Recently, only a few researchers studied acoustic cloak in the presence of flow. Since the complete study on flow effect is extremely complicated, the flow was assumed to be inviscid and steady. Two boxes with dotted lines show the comparison of the previous and present theoretical frameworks in the presence of flow: (**a**) In the previous work^7^, the flow was assumed to be *incompressible* and the differential wave operator contained the *uniform* velocity. (**b**) In the present work, the flow is *compressible* so that the sound is refracted due to density inhomogeneity and spatially varying speed of sound, and the differential wave operator contains the *non-uniform* velocity around the acoustic cloak.

As summarized in Fig. 1, early researches on the acoustic cloak have been done within a stationary medium. Recently, however, the paradigm of acoustic cloak research is shifting to analyze the effect of moving medium on sound scattering from acoustic cloak. Among only a few studies on acoustic cloak in moving medium, there was a theoretical work^7^ to investigate the effect of incompressible flow around an acoustic cloak by deriving a convective wave equation.

In 2014, Huang *et al*.^7^ formulated a convective wave equation to study an acoustic cloak in low subsonic flow. The flow was assumed to be inviscid, irrotational and incompressible flow with isentropic assumption. From the literature^7^, the equation is written by

1 $$(\frac{{D}_{{\rm{\infty }}}^{2}}{{D}_{{\rm{\infty }}}{t}^{2}}-{c}_{{\rm{\infty }}}^{2}{{\rm{\nabla }}}^{2})p^{\prime} =-\frac{{D}_{{\rm{\infty }}}}{{D}_{{\rm{\infty }}}t}({\bf{v}}\cdot {\rm{\nabla }}p^{\prime} )+{c}_{{\rm{\infty }}}^{2}{\rm{\nabla }}\cdot ({\rho }_{0}{\bf{v}}\cdot {\rm{\nabla }}{\bf{u}}^{\prime} +{\rho }_{0}{\bf{u}}^{\prime} \cdot {\rm{\nabla }}{\bf{v}}+\frac{p^{\prime} }{{c}_{{\rm{\infty }}}^{2}}{{\bf{u}}}_{0}\cdot {\rm{\nabla }}{{\bf{u}}}_{0}),$$

where *D*_∞_/*D*_∞_*t* denotes the total derivative defined by ∂/∂*t* + **u**_∞_ · ∇, **u**_∞_ is the uniform velocity, *t* is the time, *ρ* is the density, **u** is the velocity, *p* is the pressure, and ***c***_∞_ is the speed of sound. In equation (1), the fluctuating variables denoted by (·)′ depend on both time and space, and the background variables denoted by (·)_0_ are not constant but spatially varying. Here, the speed of sound ***c***_∞_ is assumed to be constant, and the differential wave operator includes the effect of uniform flow as depicted in Fig. 1a. Since the mean flow velocity **u**_0_ around an object is not uniform, they introduce a velocity difference term denoted by **v** = **u**_0_ − **u**_*∞*_ in their equivalent source term. However, as Lilley^14^ and Goldstein^15^ took account of sheared mean flow effect, more of flow effects should be contained in the left-hand-side of differential operator term, not in source term, and thus the formulation could consider physical reality. In the previous formulation^7^, the effect of non-uniform flow velocity was not coupled with the differential wave operator on the left-hand-side. In addition, it neglected the effect of compressibility which is an important factor to cause sound refraction due to density inhomogeneity.

Therefore, in this study, we propose a convective wave equation as an improved version of the previous formulation^7^ for an acoustic cloak in moving media, by (1) including the effect of non-uniform flow in differential wave operator and (2) taking the effect of compressibility into account. The mathematical derivation starts from the fluid dynamics equations for inviscid, irrotational and compressible fluid for an ideal gas and the detail derivation procedures are summarized in the Supplementary Information. Then, the convective wave equation is written as

2 $$(\frac{{D}_{0}^{2}}{{D}_{0}{t}^{2}}-{c}_{0}^{2}{\nabla }^{2})p^{\prime} ={S}_{eq}({\bf{x}},t)={S}_{comp}({\bf{x}},t)+{S}_{non}({\bf{x}},t),$$

3 $$\begin{array}{cccc}{\rm{w}}{\rm{h}}{\rm{e}}{\rm{r}}{\rm{e}} & {S}_{comp}({\bf{x}},t) & = & -{\rho }_{0}{c}_{0}^{2}[{\bf{u}}^{\prime} \cdot {\rm{\nabla }}+\frac{{D}_{0}}{{D}_{0}t}(\frac{\gamma p^{\prime} }{{\rho }_{0}{c}_{0}^{2}})+\frac{\gamma p^{\prime} }{{\rho }_{0}{c}_{0}^{2}}\frac{{D}_{0}}{{D}_{0}t}]({\rm{\nabla }}\cdot {{\bf{u}}}_{0})\\  &  &  & -{\rho }_{0}{c}_{0}^{2}\frac{{D}_{0}}{{D}_{0}t}[\frac{1}{\gamma {\rho }_{0}}({\bf{u}}^{\prime} \cdot {\rm{\nabla }}){\rho }_{0}+\frac{1}{\gamma {c}_{0}^{2}}({\bf{u}}^{\prime} \cdot {\rm{\nabla }}){c}_{0}^{2}]\,\\  &  &  & -{c}_{0}^{2}\frac{{D}_{0}p^{\prime} }{{D}_{0}t}({{\bf{u}}}_{0}\cdot {\rm{\nabla }})(\frac{1}{{c}_{0}^{2}})\\  &  &  & -{\rho }_{0}[\frac{{D}_{0}p^{\prime} }{{D}_{0}t}({{\bf{u}}}_{0}\cdot {\rm{\nabla }})-{c}_{0}^{2}{\rm{\nabla }}p^{\prime} \cdot {\rm{\nabla }}](\frac{1}{{\rho }_{0}}),\end{array}$$

4 $${S}_{non}({\bf{x}},t)={\rho }_{0}{c}_{0}^{2}{\rm{\nabla }}\cdot [2({\bf{u}}^{\prime} \cdot {\rm{\nabla }}){{\bf{u}}}_{0}+\frac{\rho ^{\prime} }{{\rho }_{0}}({{\bf{u}}}_{0}\cdot {\rm{\nabla }}){{\bf{u}}}_{0}],$$

where *D*_0_/*D*_0_*t* denotes the total derivative defined by ∂/∂*t* + **u**_0_ · ∇, *c*_0_ is the spatially varying speed of sound, and *γ* is the specific heat ratio. As shown in equation (2), the non-uniform velocity around an object is included in the differential operator. *S*_*eq*_(**x**, *t*) indicates the equivalent source due to the compressible non-uniform flow, which can be interpreted as a coupled effect of background mean flow with incident wave.

Now, we divide the mathematically abstruse terms of *S*_﻿eq_(**x**, *t*)﻿ into two parts and interpret their own physical meanings. In case that the flow is incompressible, the divergence of background velocity is zero (∇ · **u**_0_ = 0), and both density *ρ*_0_ and speed of sound *c*_0_ are constant. Therefore, the first equivalent source *S*_*comp*_(**x**, t) in equation (3) can be physically interpreted as compressibility effect because *S*_*comp*_(**x**, t) vanishes in case of incompressible flow. On the other hand, in case that the flow is uniform, it is obvious that the second equivalent source *S*_*non*_(**x**, t) in equation (4) is identically equal to zero. In other words, *S*_*non*_(**x**, t) is non-zero only for non-uniform flow, and therefore, it can be physically interpreted as non-uniformity effect. By physically dividing and defining the equivalent source terms, we may enhance the understanding of flow effect on sound scattering from an acoustic cloak and such analyses will be given in following sections.

### Scattering patterns of the existing acoustic cloak

In this section, we examine the scattering pattern of the existing convective cloak^7^ in the presence of non-uniform flow. Figure 1b shows the schematic of our problem. The monochromatic plane waves are incoming with an angle of α and propagates through the moving medium and the acoustic cloak. As shown in Fig. 1b, a circular object of radius *R*_1_ with acoustically rigid material is covered by a concentric acoustic cloak with an annulus geometry whose inner and outer radii are *R*_1_ and *R*_2_, respectively. The background medium is a compressible medium (an air with the sound speed of 334 m/s and the mass density of 1.225 kg/m^3^), and the medium is not stationary but moving with a subsonic Mach number *M*. We calculated the acoustic pressure around the cloak by solving equation (2) with finite element method (FEM).

**Figure 2:**
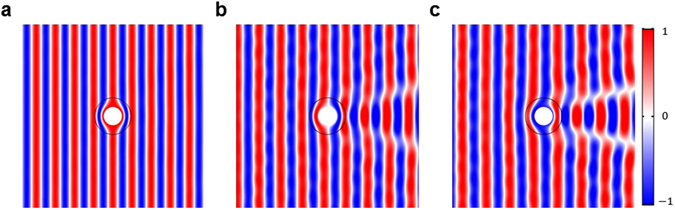
Total acoustic pressure around acoustic cloak. (**a**) The acoustic pressure around an existing cloak^1^ in stationary medium. (**b**) and (**c**) The acoustic pressure around convective cloaks^7^ in moving media with *M* = 0.1 and *M* = 0.2, respectively. For all figures, the total acoustic pressure is normalized by incident wave, *i.e*., $p^{\prime} \,/\,{p}_{{\rm{i}}{\rm{n}}{\rm{c}}}^{^{\prime} }$, and the incident angles and the Helmholtz numbers are set as *α* = 0 and *kR*_1_ = 3. As shown in (**a**), the acoustic cloak hides an object in the absence of flow and our numerical simulation shows good accuracy. On the other hand, as shown in (**b**) and (**c**), acoustic cloaks in moving media lose their hiding function as the Mach number increases, especially in forward scattering regions.

**Figure 3:**
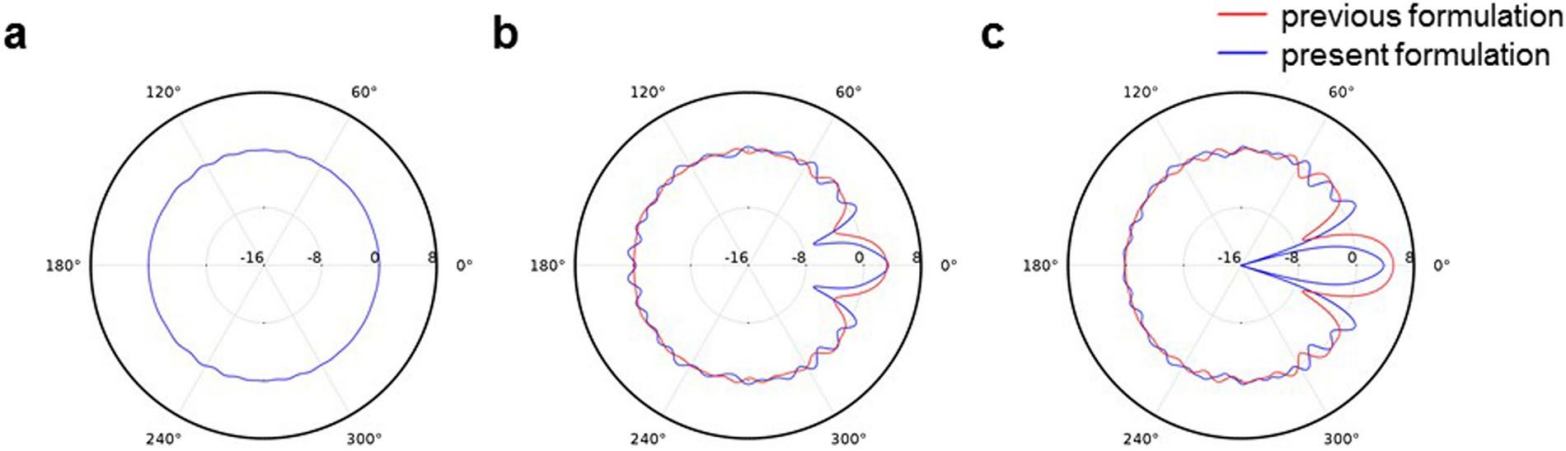
The scattering patterns of sound scattering around acoustic cloaks. (**a**) Polar plot of directivity pattern of the existing cloak^1^ in stationary medium. (**b**) and (**c**) Polar plots of directivity patterns of the convective cloaks^7^ in moving media for *M* = 0.1 and *M* = 0.2, respectively. The directivity patterns are calculated in dB scale by using equation (5) at *r* = 10*R*_1_. The incident angles and the Helmholtz numbers are set as *α* = 0 and *kR*_1_ = 3. A large deviation from 0 dB in directivity plot indicates that we have more scattering unwanted. In (**b**) and (**c**), the blue lines indicate the results of the present formulation using equation (2), and the red lines indicate the results of the previous formulation^7^ using equation (1). There is a clear difference between the scattering patterns of the previous and present formulations, and the difference becomes larger as the Mach number increases.

Figure 2a shows the acoustic pressure around a stationary cloak^1^ when *α* = 0 and *kR*_1_ = 3 in stationary medium. Figure 2b,c show the acoustic pressure around the convective cloak^7^ when α = 0 and *kR*_1_ = 3 in moving media whose Mach numbers are *M* = 0.1 and 0.2, respectively. In the contour plot, the total acoustic pressure is normalized by incident wave, *i.e*., $p^{\prime} \,/\,{p^{\prime} }_{inc}$. In Fig. 3, the directivity patterns of total acoustic pressure are calculated at *r* = 10*R*_1_ by using equation (5).

5 $$10\,{{\rm{l}}{\rm{o}}{\rm{g}}}_{10}\,\frac{{p^{\prime} }^{2}}{{p^{\prime} }_{inc}^{2}}$$

To validate our numerical method, we examined the acoustic pressure scattered from the acoustic cloak in a stationary medium. As shown in Figs 2a and 3a, the acoustic cloak makes an object acoustically invisible and our numerical solution shows good accuracy. The slight difference between the calculated directivity and the perfect circle in Fig. 3a is due to the finite number of layers used to realize the acoustic cloak. (See the **Methods**.) Thus, the difference can be removed by decreasing the thickness of each layer or equivalently by increasing the number of layers in acoustic cloak for a fixed outer r﻿adius *R*_2_.

When the Mach number is *M* = 0.1, the convective cloak fails to hide an object due to the unwanted scattering near the geometrical shadow zone as shown in Fig. 2b. Furthermore, as the Mach number increases up to *M* = 0.2, the performance of convective cloak becomes worse as shown in Fig. 2c.

Figure 3b,c show the comparison of directivity patterns between the previous^7^ and present formulations. These directivity plots say the followings: (1) it is more clearly seen that existing acoustic cloak in moving medium loses its unique property as the Mach number increases; (2) the forward scattering within the region of |*θ*| < 30° is significantly larger than other regions; (3) there is a clear difference between the present formulation and the previous formulation, and the difference becomes larger as the Mach number increases. As already discussed in the previous paper^7^, since the existing convective cloak was designed by using a simple scaling law based-on a wave equation in which there is no equivalent source term and background flow field is assumed to be uniform everywhere, such cloaking design is not adequate to hide an object submerged in non-uniform flow field.

### Comparison between the previous and present formulations

As compared in Fig. 3b,c, the numerical results obtained from two equations (1) and (2) were significantly different. In this section, the difference between the previous and present formulations will be examined in more detail.

The previous formulation, equation (1), contains the uniform wave operator on the left-hand-side of the convective wave equation, and the difference between uniform velocity and local velocity, denoted by **v**, is included in the equivalent source term^7^. One advantage to use the uniform wave operator is that the convective wave equation can be converted into the classical wave equation by using the Prandtl-Glauert transformation. And then, the analytic solution can be obtained by using Green’s function method. However, more of the real fluid effect should be included in differential operator of convective wave equation rather than in source term^13–15^. Thus, in order to consider the effect of non-uniform flow, the present formulation, equation (2), contains the non-uniform wave operator on the left-hand side of the equation and all the remaining terms are included in the equivalent source. For the purpose of physical interpretation of equivalent source terms, we decomposed them into two parts and gave their own meanings such as compressibility effect and non-uniformity effect. Through this decomposition, we can understand the mathematically abstruse equations physically.

**Figure 4:**
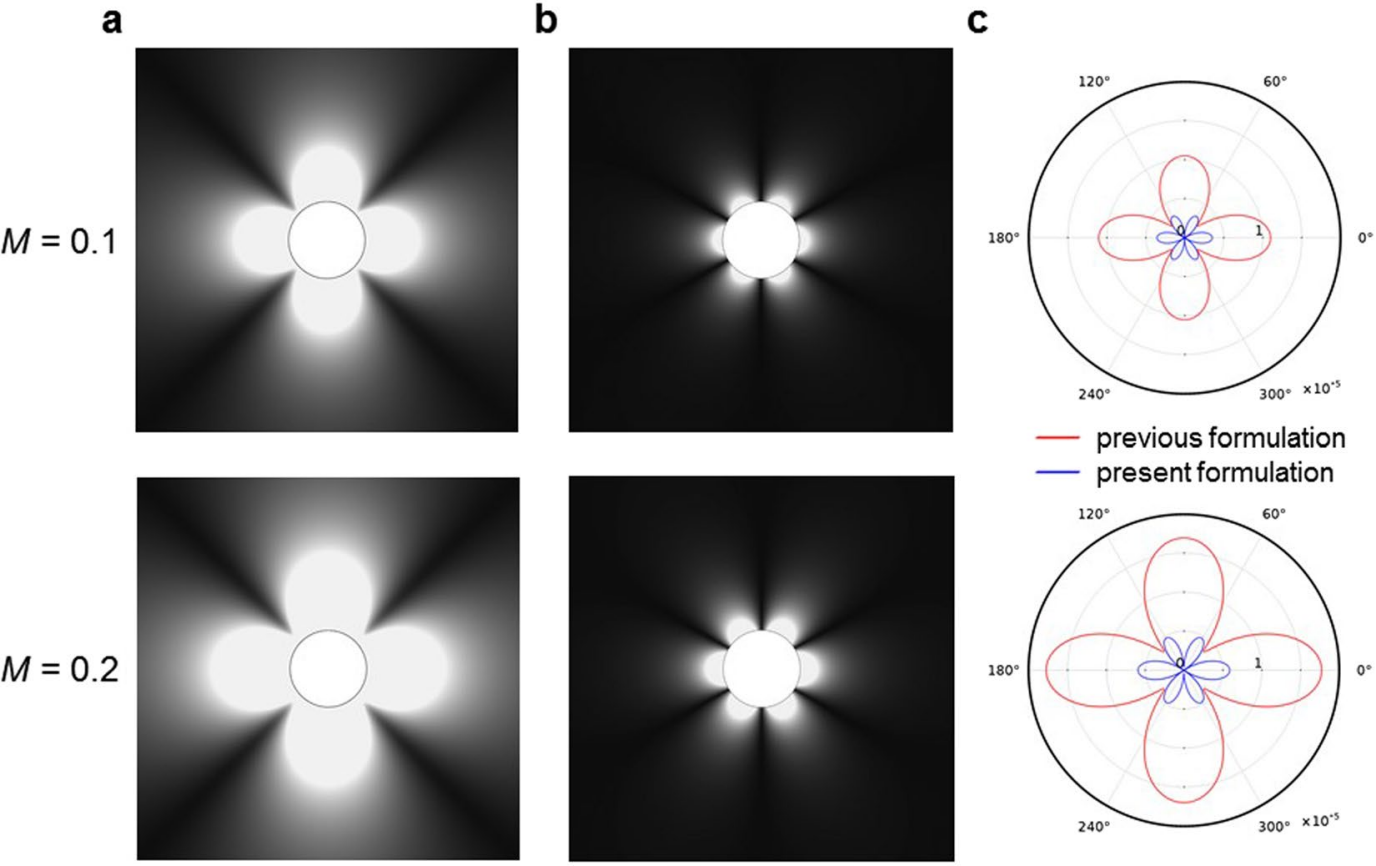
Comparison of the equivalent source terms between the previous and present formulations. (**a**) The magnitudes of equivalent source in the previous formulation^7^. (**b**) The magnitudes of equivalent source in the present formulation. (**c**) Comparison of directivity plots along the surface of the acoustic cloak (*r* = *R*_2_). In all figures, the Helmholtz numbers are set as *kR*_1_ = 3. As shown in (**a**), the magnitude of the equivalent source in the previous formulation increases as the Mach number increases and the polarity shows the quadrupole patterns. As shown in (**b**), the magnitude of the equivalent source in the present formulation also increases as the Mach number increases, and the polarity shows the hexapole patterns. As shown in (**c**), the different polarities and magnitudes of the equivalent sources are more clearly seen. The magnitude of the previous formulation is much larger than the one of the present formulation. It is because the previous formulation introduced the velocity difference term **v** = **u**_0_ − **u**_∞_ in the equivalent source term in order to make the uniform wave operator. On the other hand, the present formulation has non-uniform wave operator, and all the equivalent sources are due to the fluid compressibility and flow non-uniformity by themselves.

Now, we compare the equivalent source terms in the previous^7^ and present formulations from the view point of magnitude and polarity of the sources. Figure 4a shows the magnitudes of equivalent source in the previous formulation for *M* = 0.1 and *M* = 0.2. The magnitude of the equivalent source in the previous formulation increases as the Mach number increases whereas the polarity shows the *quadrupole* patterns regardless of the Mach numbers. Figure 4b shows the magnitudes of equivalent source in the present formulation for *M* = 0.1 and *M* = 0.2. The magnitude of the equivalent source in the present formulation also increases as the Mach number increases, and the polarity shows the *hexapole* patterns regardless of the Mach numbers.

When we compare the equivalent sources of the previous formulation^7^ and the present formulation for same Mach numbers, they have different magnitude and different polarity as shown in Fig. 4c. Here, the directivity plots show the source strength along the surface of acoustic cloak. As compared in Fig. 4c, the magnitude of the previous formulation is much larger than the one of the present formulation. It is because the previous formulation was derived from a mathematical manipulation by introducing the velocity difference term, **v** = **u**_0_ − **u**_∞_, in the equivalent source term in order to make the uniform wave operator intentionally. This velocity difference always exists when there is an object in moving medium, and **v** = **u**_0_ − **u**_∞_ increases as the Mach number increases. Thus, **v** = **u**_0_ − **u**_∞_ is a dominant factor contributing to the large magnitude of the equivalent source in the previous formulation. In contrast, the present formulation has non-uniform wave operator without any intentional mathematical manipulation, and thus, all the equivalent sources are due to the fluid compressibility and flow non-uniformity by themselves.

Identifying the characteristics of equivalent sources in convective wave equation is very important in the study of acoustic cloak within flow. Firstly, we can predict the change of scattering pattern of acoustic cloak in the presence of flow and understand the underlying physics of flow-induced sound source due to the acoustic cloak. Secondly, based-on the physical understanding, we can design a better acoustic cloak within non-uniform flow not by an experience (or an intuition) but by a systematical and logical approach.

### Comparison between compressibility and non-uniformity effects

**Figure 5:**
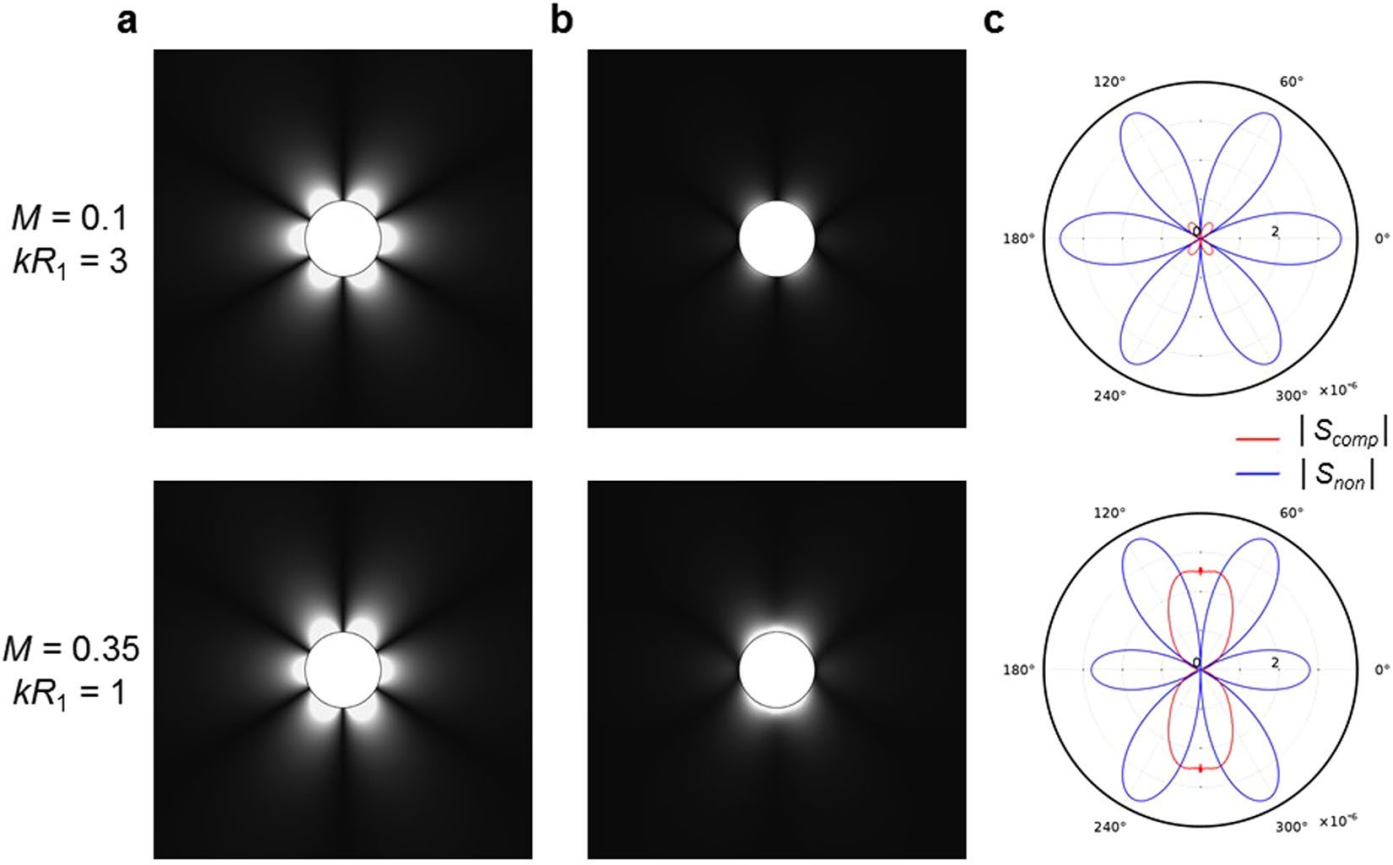
Comparison between compressibility and non-uniformity effects. (**a**) The magnitude of non-uniformity source terms in equation (4). (**b**) The magnitude of compressibility source terms in equation (3). (**c**) Comparison of directivity plots along the surface of the acoustic cloak (*r* =*R*_2_). In all figures, the Mach numbers and the Helmholtz numbers are set as *kR*_1_ = 3 and *M* = 0.1 for top panel and *kR*_1_ = 1 and *M* = 0.35 for bottom panel. As shown in (**a**) and (**b**), the non-uniformity term shows the hexapole pattern regardless of the Helmholtz numbers and the Mach numbers, but the polarity and magnitude of compressibility term depend on the Helmholtz number and the Mach number. As shown in (**c**), the polarity of compressibility term is changed from skewed quadrupole to dipole as the Mach number increases and the Helmholtz number decreases.

In this section, the equivalent source terms of the present formulation are investigated qualitatively as well as quantitatively. The equivalent source terms, *S*_*eq*_(**x**, *t*), in the present formulation describe the coupled effect of background flow with incident acoustic wave. As explained in theoretical framework section, the equivalent source terms are divided into *S*_*non*_ and *S*_*comp*_ in the present formulation. In case of incompressible flow, *S*_*comp*_ was zero since divergence of velocity and gradient of density are zero (∇ · **u**_0_ = 0 and ∇*ρ*_0_ = **0**). However, since we are considering the compressible flow, *S*_*comp*_ should be considered as well. Figure 5a,b show the magnitudes of equivalent source terms (|*S*_*non*_| and |*S*_*comp*_|) for *M* = 0.1 and *kR*_1_ = 3, and *M* = 0.35 and *kR*_1_ = 1, respectively. The directivity plots along the surface of the acoustic cloak (*r* = *R*_2_) are shown in Fig. 5c for the comparison of magnitude and polarity in each equivalent source term.

From top panel of Fig. 5 for *M* = 0.1 and *kR*_1_ = 3, we find the following characteristics of our equivalent source: (i) the non-uniformity term shows the hexapole pattern, (ii) the compressibility term shows the skewed-quadrupole pattern, and (iii) the magnitude of non-uniformity term is much larger than the one of compressibility term. However, from the bottom panel of Fig. 5 for *M* = 0.35 and *kR*_1_ = 1, it is observed that (i) the non-uniformity term shows the hexapole pattern, (ii) the compressibility term shows the dipole pattern, (iii) the magnitude of compressibility is not negligible. From the Fig. 5, we find that the non-uniformity term shows the hexapole pattern regardless of the Helmholtz numbers and the Mach numbers, but the polarity and magnitude of compressibility term strongly depend on the Helmholtz number and the Mach number. The skewed quadrupole is dominant for low Mach number and high Helmholtz number. However, as the Mach number increases and the Helmholtz number decreases, the dominant polarity is shifted from the skewed quadrupole to the dipole.

**Figure 6:**
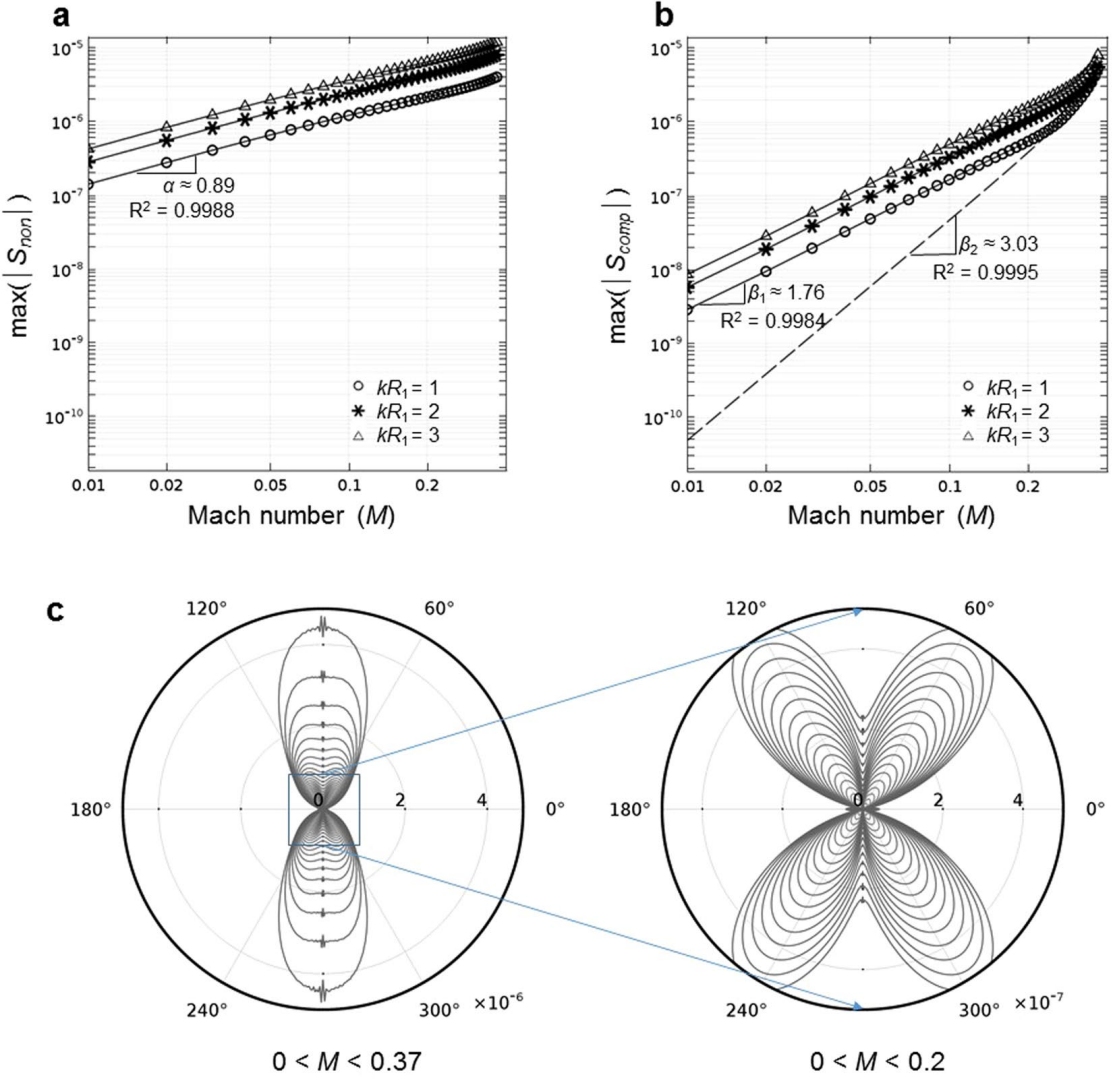
Parametric study on velocity law of the magnitude in compressibility and non-uniformity source terms. (**a**) The log-log plot of maximum magnitude of non-uniformity source term *S*_*non*_ versus Mach number for *kR*_1_ = 1, 2, 3. (**b**) The log-log plot of maximal magnitude of compressibility source term *S*_*comp*_ versus Mach number for *kR*_1_ = 1, 2, 3. (**c**) The directivity plots of compressibility source term *S*_*comp*_ along the surface of acoustic cloak for *kR*_1_ = 1 and various Mach numbers. In all figures, the Mach number is changing from 0.01 to 0.37. In (**b**), symbols (circles, asterisks, and triangles) indicate the maxima of compressibility source terms for *kR*_1_ = 1, 2, 3, respectively. The dashed line indicates maximum values of dipole strength. In (**c**), whereas the left panel shows the directivity plot for all Mach numbers from 0.01 to 0.37, the right panel shows the enlarged directivity plot for relatively low Mach numbers from 0.01 to 0.2 in order to observe the transition of polarity type from quadrupole to dipole as the Mach number increases.

For a quantitative investigation on the polarity and the magnitude of each source term, a simple parametric study was performed for various Mach numbers and Helmholtz numbers. Figure 6a,b show the log-log plot of maximum magnitude in directivity versus Mach number for *kR*_1_ = 1, 2, 3. Here, the Mach number varies from 0.01 to 0.37 with an increment of 0.01 (37 data points).

Figure 6a shows the relation between the Mach number and the source strength due to the non-uniformity term. It is shown that the maximum magnitude in hexapole directivity increases as the Mach number increases regardless of the Helmholtz numbers. There is a power-law relation, *max*(|*S*_*non*_|) ∝ *M*^0.9^, between the Mach number and the maximum magnitude in directivity p﻿lots. Roughly speaking, the equivalent source strength due to the non-uniform flow is nearly proportional to the Mach number.

Figure 6b shows the relation between the Mach number and the source strength due to the compressibility term. In this case, the strength should be divided into two types of polarity: the one is the skewed-quadrupole and the other is the dipole because the dominant polarity type shifts from the skewed-quadrupole to the dipole as shown in Fig. 6c. In case of low subsonic flow (*M* < 0.2), the dominant pattern is the skewed quadrupole and there’s a power-law relation, *max*(|*S*_*comp*_|) ∝ *M*^1.8^, between the Mach number and the maximum magnitude. The equivalent source strength due to the fluid compressibility is nearly proportional to the square of Mach number. However, in case of relatively high subsonic flow (*M* > 0.3), the skewed-quadrupole is not dominant anymore and the dipole has comparable or larger magnitude than the skewed-quadrupole. When we separate the dipole strength and find a relation between the maximum magnitude of dipole and the Mach number, we obtain another power-law such as *max*(|*S*_*comp*_|) ∝ *M*^3.0^. Although the compressibility effect is much smaller than non-uniformity effect in low Mach number range, it is not negligible in high Mach number range, especially for small Helmholtz number. Here’s the summary of power-law relation between the Mach number and the maximum strength of the equivalent source term due to the flow non-uniformity and the fluid compressibility.

6 $$max(|{S}_{non}|)\propto {M}^{0.9},\,{\rm{hexapole}}\,{\rm{type}}$$

7 $$max(|{S}_{comp}|)\propto \{\begin{array}{l}{M}^{1.8},\,{\rm{quadrupole}}\,{\rm{type}}\\ {M}^{3.0},\,{\rm{dipole}}\,{\rm{type}}\end{array}$$

Concluding the results, we emphasize the importance of compressible flow analysis for both identifying the equivalent source terms of acoustic cloak (or metamaterials) in high subsonic flow and designing a new acoustic cloak within flow.

## Discussion

In order to improve the physical reality of acoustic cloak in moving medium, we need to consider the more of real fluid effects such as viscosity and shear refraction (sound refraction due to sheared mean flow). Viscosity is an important factor especially in a low Mach number regime because self-induced noise can be generated due to the flow separation. As for the shear refraction effect, Lilley^14^ and Goldstein^15^ pointed out that a differential operator that neglects the shear refraction term is not strictly correct in case that the refraction of short waves occurs through the shear layer. The effect of shear refraction can be included in our formulation by modifying it into the Lilley’s wave operator^14^ which is a third order differential operator.

Before considering all the fluid effects with physical reality, sometimes it is useful to analyze a simple mathematical model, by which we can understand the underlying physics theoretically. Therefore, instead of solving the Navier-Stokes equation directly, we analyzed the steady potential flow. Since these have not been considered in acoustic cloak research yet, we focused on the effects of compressibility and non-uniformity. In addition, we emphasized the physical interpretations of mathematically abstruse source terms in our formulation. By performing numerical simulations based on the present framework, we analyzed (1) the effect of potential flow around an acoustic cloak on the sound scattering pattern, (2) the difference between the previous and present frameworks, and (3) the physical properties of each term in the equivalent sources quantitatively as well as qualitatively.

**Figure 7:**
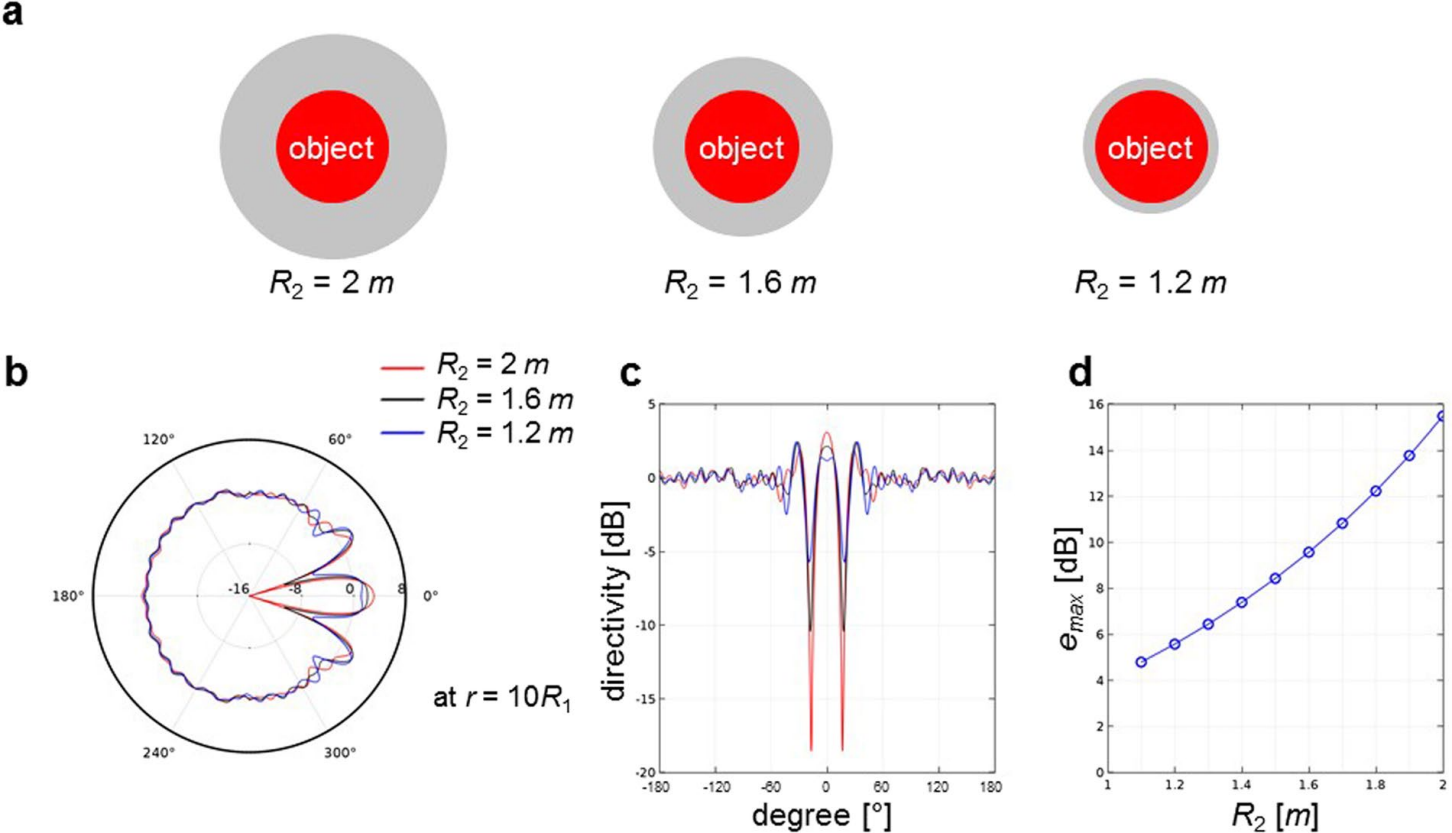
Effect of thickness of the acoustic cloak on scattering pattern. (**a**) Schematics of acoustic cloaks with reduced thickness for *R*_2_ = 2, 1.6, and 1.2 *m*. (**b**) Polar plot of directivity patterns of the acoustic cloaks with reduced thickness in moving medium for *M* = 0.2. (**c**) Rectangular plot of directivity patterns of the acoustic cloaks with reduced thickness in moving medium for *M* = 0.2. The directivity patterns are calculated in dB scale by using equation (5) at *r* = 10*R*_1_. (**d**) Maximal errors calculated by using equation (8) at *r* = 10*R*_1_ for various thicknesses of acoustic cloak. As shown in (**b**) and (**c**), the thinner cloak shows less of scattering from the cloak in moving medium. In addition, as clearly shown in (**d**), the scattering decreases as the thickness of acoustic cloak decreases.

Even if this paper is aiming at developing an improved mathematical model and performing numerical simulations, we tried to make use of our model for predicting the acoustic scattering from newly designed cloaks as a practical application. There are at least two approaches to reduce the scattering from an acoustic cloak in moving medium. One is to make the acoustic cloak thinner, and the other is to modify the material properties of the cloak. The effect of thickness of the cloak is critical in moving medium whereas it is not in a stationary medium. We performed a parametric study on the thickness of acoustic cloak by changing it from *R*_1_ to 0.2*R*_1_, and then compared the cloaking performance for *M* = 0.2. (See the Fig. 7a). The cloaking performance was evaluated by *e*_*max*_ as defined in equation (8).

8 $${e}_{max}=\begin{array}{c}max\\ 0\le \theta  < 2\pi \end{array}|10\,{{\rm{l}}{\rm{o}}{\rm{g}}}_{10}\,\frac{{p^{\prime} }^{2}}{{p^{\prime} }_{inc}^{2}}|\,{\rm{a}}{\rm{t}}\,r=10{R}_{1}$$

As shown in Fig. 7b,c, the thinner cloak shows less of unwanted scattering from the cloak in moving medium. In addition, as clearly shown in Fig. 7d, the scattering decreases as the thickness of acoustic cloak decreases. It is because the thinner cloak reduces the magnitudes of velocity gradient and density inhomogeneity around the cloak, which play an important role in the wave operator as well as the equivalent source terms.

**Figure 8:**
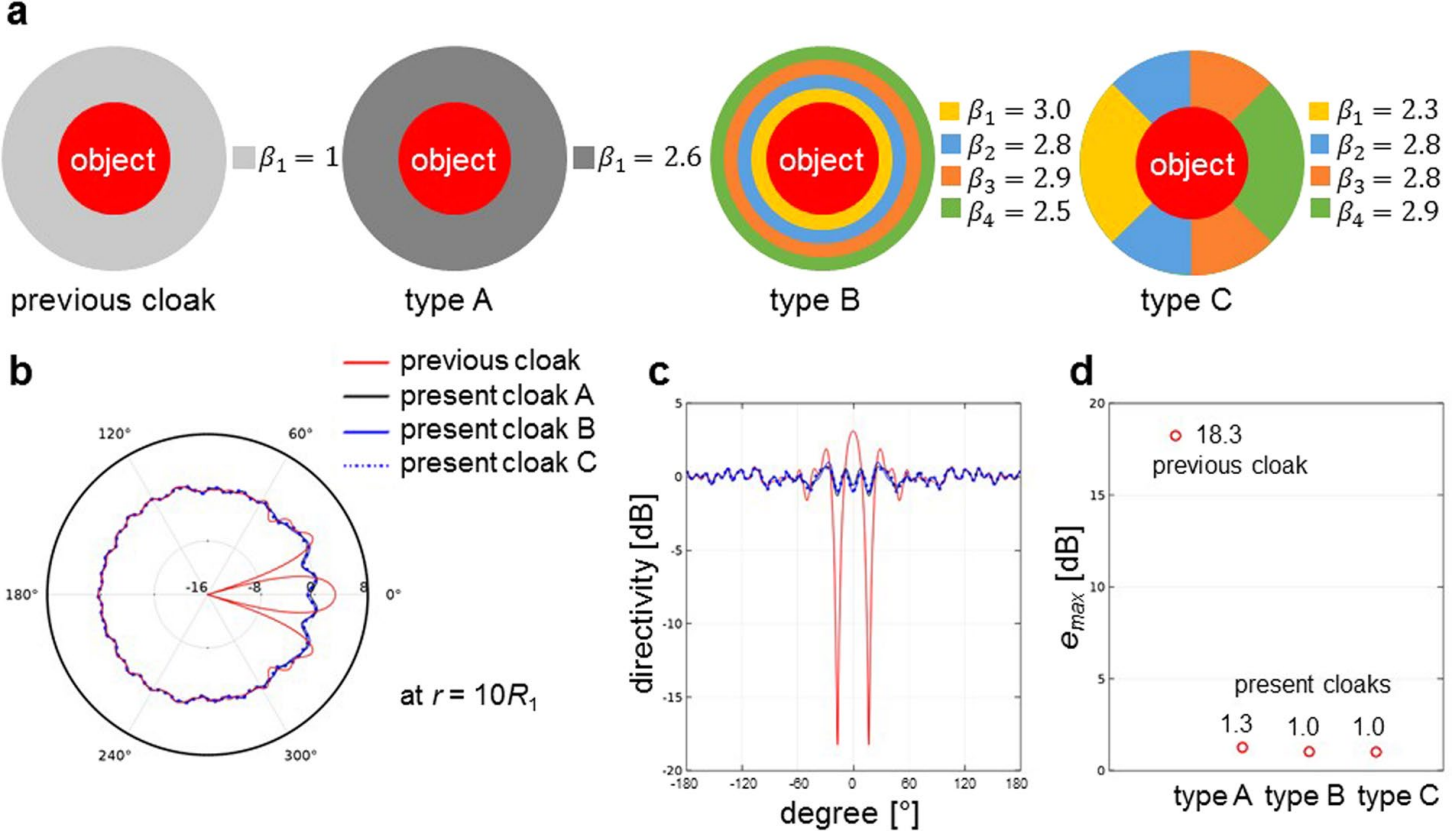
Shapes, material properties, and performances of newly designed convective cloaks. (**a**) Schematics of acoustic cloaks with modified material properties as shown in equation (9). Type A shows a single-zone modification (*N* = 1) with optimized value of *β*_1_, and types B and C show multiple-zone modifications (*N* = 4) with optimized values of *β*_*i*_ for *i* = 1, 2, 3, 4. (**b**) Polar plot of directivity patterns of the previous and present cloaks in moving medium for *M* = 0.2. (**c**) Rectangular plot of directivity patterns of the previous and present cloaks in moving medium for *M* = 0.2. The directivity patterns are calculated in dB scale by using equation (5) at *r* = 10*R*_1_. (**d**) Maximal errors calculated by using equation (8) at *r* = 10*R*_1_ for the previous and present cloaks. As shown in (**d**), three types of acoustic cloaks show better performance compared to the existing convective cloak^7^.

In order to achieve a better performance of the acoustic cloak in moving medium than the thinner acoustic cloaks, we propose three types of newly designed convective cloaks by modifying the material properties of a previous convective cloak. As shown in Fig. 8a. the annulus type of acoustic cloak is divided into a single zone or multiple zones. When an acoustic cloak is divided into *N* zones, the modified material properties in each zone are given as follows:

9 $$\begin{array}{rcl}\frac{{\rho }_{i}^{r}(r)}{{\rho }_{0}} & = & \frac{r}{r-{R}_{1}}\frac{1}{1+{\beta }_{i}M\,\cos \,\alpha },\\ \frac{{\rho }_{i}^{\theta }(r)}{{\rho }_{0}} & = & \frac{r-{R}_{1}}{r}\frac{1}{1+{\beta }_{i}M\,\cos \,\alpha },\\ \frac{{\kappa }_{i}(r)}{\,{\kappa }_{0}} & = & \,{(\frac{{R}_{2}-{R}_{1}}{{R}_{2}})}^{2}\frac{r}{r-{R}_{1}}(1+{\beta }_{i}M\,\cos \,\alpha ),\\ for\,i & = & 1,2,\ldots ,N,\end{array}$$

where *β*_*i*_ indicates a design parameter to modify the material properties of *i*-th zone and *N* is the number of multiple zones. For type A (single-zone modification), material properties in case of *β*_1_ = 0 are exactly same with those of the stationary cloak^1^, and material properties in case of *β*_1_ = 1 correspond to those of the previous convective cloak^7^. By minimizing *e*_*max*_, the optimized value of *β*_1_ was obtained by *β*_1_ = 2.6 for type A with a single zone modification. Here, the minimized *e*_*max*_ was approximately 1.3 dB. For types B and C (multiple zone modification), the optimized values of the design parameter *β*_*i*_ (for *i* = 1, 2, 3, 4) for each zone are listed in Fig. 8a with a minimized scattering of *e*_*max*_ = 1.0 dB. As shown in Fig. 8a,b,c, three types of acoustic cloaks show far better performance compared to the existing convective cloak. Further optimization with thinner thickness is expected to yield an almost perfect cloak for a given Mach number and an incidence angle. However, it’s still a challenging problem to design a convective cloak in moving medium for arbitrary Mach numbers and incidence angles, and it is our on-going research topic.

## Methods

### Cloaking Metamaterials

In most of acoustic cloak research^1–4^, the acoustic cloak covering an object was designed adequate to a stationary medium. However, such a cloak fails to hide an object in the presence of flow^6–9^. Thus, by using an analogue transformation method^6^, a design of convective cloak was proposed as equation (10) in a recent work^7^.

10 $$\begin{array}{rcl}\frac{{\rho }^{r}(r)}{{\rho }_{0}} & = & \frac{r}{r-{R}_{1}}\frac{1}{1+M\,\cos \,\alpha },\\ \frac{{\rho }^{\theta }(r)}{{\rho }_{0}} & = & \,\frac{r-{R}_{1}}{r}\frac{1}{1+M\,\cos \,\alpha },\\ \frac{\kappa (r)}{\,{\kappa }_{0}} & = & {(\frac{{R}_{2}-{R}_{1}}{{R}_{2}})}^{2}\frac{r}{r-{R}_{1}}(1+M\,\cos \,\alpha ).\end{array}$$

Here, *κ* is the bulk modulus, *M* (=|**u**_∞_|/*c*_0_) is the Mach number, and the superscripts of (·)^*r*^ and (·)^*θ*^ represent the anisotropic properties of the acoustic cloak in *r* and *θ* directions, respectively. For a practical implementation of anisotropic properties in numerical analysis, we used a multi-layered structure in which each layer has homogeneous physical properties^16^. In this work, we used 50 layers of the multi-layered structure, which are sufficient to realize the anisotropic material properties of the acoustic cloak. The thickness of each layer in the multi-layered structure is much shorter than the wavelength of incoming plane wave in order to satisfy the homogenization limit.

### Governing Equations and Boundary Conditions

The background medium is moving with a subsonic Mach number *M* and the flow is assumed to be steady potential flow. The background variables can be obtained by solving the following equations derived from equations (S4)–(S6) and (S10) in the Supplementary Information.

11 $$({{\bf{u}}}_{0}\cdot \nabla ){\rho }_{0}+{\rho }_{0}\nabla \cdot {{\bf{u}}}_{0}=0,$$

12 $$\frac{1}{2}\nabla {|{{\bf{u}}}_{0}|}^{2}+\frac{\gamma }{\gamma -1}\frac{{p}_{ref}}{{\rho }_{ref}^{\gamma }}\nabla ({\rho }_{0}^{\gamma -1})={\bf{0}},$$

where *p*_ref_ and *ρ*_ref_ are the ambient pressure and density. The streamlines of the potential flow are illustrated by gray lines in Fig. 1b. The boundary condition is imposed by impermeable slip wall condition at *r* = *R*_2_.

Since there is no flow inside the acoustic cloak, the governing equation of sound is a classical wave equation, whereas the governing equation of sound in the moving fluid is equation (2). Since the background flow is assumed to be potential flow, the acoustic pressure and fluid velocity are set to be continuous at the interface between the moving fluid and the acoustic cloak as adopted in the previous work^7^. The impermeability boundary condition is imposed on *r* = *R*_1_ since the circular object is an acoustically rigid material.

### Numerical Simulations

We calculated the acoustic pressure around the cloak by solving equation (2) with finite element method (FEM). Since the equivalent source terms also contain the unknown variables such as *p*′ and **u**′, we used the iterative method with the initial values calculated from the first Born approximation^17^ for the purpose of fast convergence. Then, the total pressure field was obtained by iteratively solving equation (2) until the relative error is less than 0.05%.

The size of computational domain was [−11(m),11(m)] × [−11(m),11(m)]. We used triangular mesh for background medium and quadrilateral mesh for cloaking material. Total number of numerical elements was 110,000 and the element size was determined to satisfy 10 nodes per wavelength. To prevent unphysical reflections at the boundary of the computational domain, the buffer zone was used by creating an extra domain in which the amplitude of out-going wave is artificially damped^18^.

## Electronic supplementary material

Supplementary Information
