# Author Correction: Effect of compressibility and non-uniformity in flow on the scattering pattern of acoustic cloak

**DOI:** 10.1038/s41598-018-23235-3

**Published:** 2018-04-19

**Authors:** Hyeonbin Ryoo, Wonju Jeon

**Affiliations:** Department of Mechanical Engineering, Korea Advanced Institute of Science and Technology, Daejeon, 34141 Korea

Correction to: *Scientific Reports* 10.1038/s41598-017-02143-y, published online 18 May 2017

This Article contains typographical errors in the Supplementary Information file.

In Equation S1,

$$\frac{D\rho }{Dt}+\nabla \cdot (\rho {\bf{u}})=0,$$

should read:

$$\frac{D\rho }{Dt}+\rho \nabla \cdot {\bf{u}}=0,$$

In Equation S8,

$$\frac{{D}_{0}{\bf{u}}^{\prime} }{{D}_{0}t}+({\bf{u}}^{\prime} \cdot \nabla ){{\bf{u}}}_{0}+\frac{\rho ^{\prime} }{{\rho }_{0}^{2}}\nabla {p}_{0}+\frac{1}{{\rho }_{0}}\nabla p^{\prime} =0,$$

should read:

$$\frac{{D}_{0}{\bf{u}}^{\prime} }{{D}_{0}t}+({\bf{u}}^{\prime} \cdot \nabla ){{\bf{u}}}_{0}-\frac{\rho ^{\prime} }{{\rho }_{0}^{2}}\nabla {p}_{0}+\frac{1}{{\rho }_{0}}\nabla p^{\prime} =0,$$

In Equation S13,

$$\frac{{D}_{0}{\bf{u}}^{\prime} }{{D}_{0}t}+\frac{1}{{\rho }_{0}}\nabla p^{\prime} =-({\bf{u}}^{\prime} \cdot \nabla ){{\bf{u}}}_{0}-\frac{\rho ^{\prime} }{{\rho }_{0}^{2}}\nabla {p}_{0}.$$

should read:

$$\frac{{D}_{0}{\bf{u}}^{\prime} }{{D}_{0}t}+\frac{1}{{\rho }_{0}}\nabla p^{\prime} =-({\bf{u}}^{\prime} \cdot \nabla ){{\bf{u}}}_{0}+\frac{\rho ^{\prime} }{{\rho }_{0}^{2}}\nabla {p}_{0}.$$

And finally,

‘By using equation (S5), $\nabla {p}_{0}/{\rho }_{0}$ in the second term on RHS of equation (S13) is replaced by $({{\bf{u}}}_{0}\cdot \nabla ){{\bf{u}}}_{0}$.’

Should read:

‘By using equation (S5), $\nabla {p}_{0}/{\rho }_{0}$ in the second term on RHS of equation (S13) is replaced by $-({{\bf{u}}}_{0}\cdot \nabla ){{\bf{u}}}_{0}$.’

