# Supplementary material for: Effect of compressibility and non-uniformity in flow on the scattering pattern of acoustic cloak
Source: Sci Rep. 2017 May 18;7:2125. doi: 10.1038/s41598-017-02143-y (PMC5437017; doi:10.1038/s41598-017-02143-y)
Supplement: Supplementary file 1 — Supplementary Information [file 41598_2017_2143_MOESM1_ESM.pdf]

Supplementary Information

Effect of compressibility and non-uniformity in flow on the scattering pattern of acoustic cloak

Hyeonbin Ryoo[1], Wonju Jeon[1,*]

[1]Department of Mechanical Engineering, Korea Advanced Institute of Science and Technology, Daejeon 34141, Korea

*To whom correspondence should be addressed: wonju.jeon@kaist.ac.kr

Derivation of Equation (2)

Equation (2) of the main article has the convective wave operator on LHS and the equivalent source terms on RHS by following the similar mathematical procedure to the Lighthill's acoustic analogy[1]. Our derivation starts from the governing equations of inviscid, irrotational and compressible flow.

$$\frac{D\rho}{Dt} + \nabla \cdot (\rho \mathbf{u}) = 0, \tag{S1}$$

$$\frac{D\mathbf{u}}{Dt} + \frac{1}{\rho}\nabla p = \mathbf{0}, \tag{S2}$$

$$\frac{Ds}{Dt} = 0, \tag{S3}$$

where D/Dt denotes the total derivative defined by $\partial/\partial t + \mathbf{u} \cdot \nabla$, and s is the entropy. Since we are interested in the perturbed quantities in physical variables, we divide each variable of the equations (S1)–(S3) into fluctuating and background variables as the following form: $\rho = \rho_0 + \rho'$, $\mathbf{u} = \mathbf{u}_0 + \mathbf{u}'$, $p = p_0 + p'$, and $s = s_0 + s'$. The fluctuating variables are assumed to be much smaller than the background variables, that is, $\rho' \ll \rho_0$, $|\mathbf{u}'| \ll |\mathbf{u_0}|$, $p' \ll p_0$, and $s' \ll s_0$. In addition, since the flow is assumed to be steady, the background variables are independent of time, that is, $\partial\rho_0/\partial t = 0$, $\partial\mathbf{u}_0/\partial t = \mathbf{0}$ and $\partial s_0/\partial t = 0$. Based on these assumptions, equations (S1)–(S3) are linearized and recast into the following six equations.

$$(\mathbf{u}_0 \cdot \nabla)\rho_0 + \rho_0 \nabla \cdot \mathbf{u}_0 = 0, \tag{S4}$$

$$(\mathbf{u}_0 \cdot \nabla)\mathbf{u}_0 + \frac{1}{\rho_0}\nabla p_0 = \mathbf{0}, \tag{S5}$$

$$(\mathbf{u}_0 \cdot \nabla)s_0 = 0, \tag{S6}$$

$$\frac{D_0\rho'}{D_0 t} + (\mathbf{u}' \cdot \nabla)\rho_0 + \rho'\nabla \cdot \mathbf{u_0} + \rho_0 \nabla \cdot \mathbf{u}' = 0, \tag{S7}$$

$$\frac{D_0\mathbf{u}'}{D_0 t} + (\mathbf{u}' \cdot \nabla)\mathbf{u_0} + \frac{\rho'}{\rho_0^2}\nabla p_0 + \frac{1}{\rho_0}\nabla p' = \mathbf{0}, \tag{S8}$$

$$\frac{D_0 s'}{D_0 t} + (\mathbf{u}' \cdot \nabla)s_0 = 0. \tag{S9}$$

Since the medium is assumed to be an ideal gas, the state equation is written as

$$p = p_{ref}\left(\frac{\rho}{\rho_{ref}}\right)^{\gamma} \exp\left[\frac{\gamma - 1}{R}\left(s - s_{ref}\right)\right], \tag{S10}$$

where R is the gas constant for air at standard temperature and pressure, and $(\cdot)_{ref}$ denotes the equilibrium values of pressure, density and entropy. For the fluctuating variables, the state equation is given as

$$p' = c_0^2\rho' + hs', \tag{S11}$$

where c_0 is the speed of sound defined by $\sqrt{\partial p_0/\partial\rho_0} = \sqrt{\gamma p_0/\rho_0}$, and h denotes $\partial p_0/\partial s_0 = (\gamma - 1)p_0/R$. Equations (S7)-(S9) are rewritten by a set of two coupled equations in terms of p' and $\mathbf{u}'$ by using the equations (S10) and (S11)

$$\frac{1}{\rho_0 c_0^2}\frac{D_0 p'}{D_0 t} + \nabla\cdot\mathbf{u}' = -\frac{1}{\rho_0 c_0^2}(\mathbf{u}'\cdot\nabla)p_0 - \frac{\gamma p'}{\rho_0 c_0^2}\nabla\cdot\mathbf{u}_0, \tag{S12}$$

$$\frac{D_0\mathbf{u}'}{D_0 t} + \frac{1}{\rho_0}\nabla p' = -(\mathbf{u}'\cdot\nabla)\mathbf{u}_0 - \frac{\rho'}{\rho_0^2}\nabla p_0. \tag{S13}$$

The derivation of equations (S12) and (S13) can also be found in the precedent literature[2]. But they omitted the terms ∇p_0 and $\nabla\cdot\mathbf{u}_0$ when deriving a wave equation because the terms are order of $|\mathbf{u}_0|^2/c_0^2$, which is small enough in the range of $M < 0.1$. However, in this work, since we are also taking account of high subsonic regime, these terms are not negligible.

Using the identity of $\rho_0 c_0^2 = \gamma p_0$ from the definition of speed of sound, $(\mathbf{u}'\cdot\nabla)p_0$ in the first term on RHS of equation (S12) is replaced by $(\mathbf{u}'\cdot\nabla)(\rho_0 c_0^2/\gamma)$. By using equation (S5), $\nabla p_0/\rho_0$ in the second term on RHS of equation (S13) is replaced by $(\mathbf{u}_0\cdot\nabla)\mathbf{u}_0$. Then we have the following equations,

$$\frac{1}{\rho_0 c_0^2}\frac{D_0 p'}{D_0 t} + \nabla\cdot\mathbf{u}' = -\frac{1}{\gamma\rho_0 c_0^2}(\mathbf{u}'\cdot\nabla)(\rho_0 c_0^2) - \frac{\gamma p'}{\rho_0 c_0^2}\nabla\cdot\mathbf{u}_0, \tag{S14}$$

$$\frac{D_0\mathbf{u}'}{D_0 t} + \frac{1}{\rho_0}\nabla p' = -(\mathbf{u}'\cdot\nabla)\mathbf{u}_0 - \frac{\rho'}{\rho_0}(\mathbf{u}_0\cdot\nabla)\mathbf{u}_0. \tag{S15}$$

To derive a convective wave equation only for p', we take the operators $D_0/D_0 t$ on the both side of equation (S14) and $\nabla\cdot$ on the both side of equation (S15).

$$\frac{D_0}{D_0 t}\left(\frac{1}{\rho_0 c_0^2}\frac{D_0 p'}{D_0 t}\right) + \frac{D_0}{D_0 t}(\nabla\cdot\mathbf{u}') = \frac{D_0}{D_0 t}\left[-\frac{1}{\gamma\rho_0 c_0^2}(\mathbf{u}'\cdot\nabla)(\rho_0 c_0^2) - \frac{\gamma p'}{\rho_0 c_0^2}\nabla\cdot\mathbf{u}_0\right], \tag{S16}$$

$$\nabla\cdot\left(\frac{1}{\rho_0}\nabla p'\right) + \nabla\cdot\left(\frac{D_0\mathbf{u}'}{D_0 t}\right) = \nabla\cdot\left[-(\mathbf{u}'\cdot\nabla)\mathbf{u}_0 - \frac{\rho'}{\rho_0}(\mathbf{u}_0\cdot\nabla)\mathbf{u}_0\right]. \tag{S17}$$

After using an identity of

$$\frac{D_0}{D_0 t}(\nabla\cdot\mathbf{u}') - \nabla\cdot\left(\frac{D_0\mathbf{u}'}{D_0 t}\right) = -\frac{\partial u_{0i}}{\partial x_j}\frac{\partial u_j'}{\partial x_i} = -\nabla\cdot[(\mathbf{u}'\cdot\nabla)\mathbf{u}_0] + (\mathbf{u}'\cdot\nabla)(\nabla\cdot\mathbf{u}_0), \tag{S18}$$

where u_{0i}, x_i and u_i' are the i-th components of $\mathbf{u_0}$, $\mathbf{x}$ and $\mathbf{u}'$ for $i,j = 1,2,3$, subtracting equation (S16) from equation (S17) and multiplying $\rho_0 c_0^2$ on both sides yield the following equations,

$$\rho_0 c_0^2\frac{D_0}{D_0 t}\left(\frac{1}{\rho_0 c_0^2}\frac{D_0 p'}{D_0 t}\right) - \rho_0 c_0^2\nabla\cdot\left(\frac{\nabla p'}{\rho_0}\right) = S_1(\mathbf{x},t) + S_2(\mathbf{x},t), \tag{S19}$$

where

$$S_1(\mathbf{x},t) = \rho_0 c_0^2\nabla\cdot\left[2(\mathbf{u}'\cdot\nabla)\mathbf{u}_0 + \frac{\rho'}{\rho_0}(\mathbf{u}_0\cdot\nabla)\mathbf{u}_0\right], \tag{S20}$$

$$\begin{aligned} S_2(\mathbf{x},t) = &-\rho_0 c_0^2\left[\mathbf{u}'\cdot\nabla + \frac{D_0}{D_0 t}\left(\frac{\gamma p'}{\rho_0 c_0^2}\right) + \frac{\gamma p'}{\rho_0 c_0^2}\frac{D_0}{D_0 t}\right](\nabla\cdot\mathbf{u}_0) \\ &-\rho_0 c_0^2\frac{D_0}{D_0 t}\left[\frac{1}{\gamma\rho_0}(\mathbf{u}'\cdot\nabla)\rho_0 + \frac{1}{\gamma c_0^2}(\mathbf{u}'\cdot\nabla)c_0^2\right]. \end{aligned} \tag{S21}$$

The equivalent source terms are due to the compressible non-uniform flow, and such source terms are the coupled effect of background flow with acoustic perturbation. Since the background density and the speed of sound are not temporally varying but spatially varying, the equation (S19) can be rewritten as

$$\frac{D_0^2 p'}{D_0 t^2} - c_0^2 \nabla^2 p' = S_1(\mathbf{x}, t) + S_2(\mathbf{x}, t) + S_3(\mathbf{x}, t) + S_4(\mathbf{x}, t), \tag{S22}$$

where

$$S_3(\mathbf{x}, t) = -c_0^2 \frac{D_0 p'}{D_0 t} (\mathbf{u_0} \cdot \nabla) \left(\frac{1}{c_0^2}\right), \tag{S23}$$

$$S_4(\mathbf{x}, t) = -\rho_0 \left[\frac{D_0 p'}{D_0 t} (\mathbf{u_0} \cdot \nabla) - c_0^2 \nabla p' \cdot \nabla\right] \left(\frac{1}{\rho_0}\right). \tag{S24}$$

The additional source terms, S_3 and S_4, cause sound refraction due to the spatially varying speed of sound and the density inhomogeneity, respectively. In case that the background flow is incompressible, the terms S_2, S_3 and S_4 are zeros and they are coined by compressibility term denoted as S_{comp}. Similarly, in case of uniform flow, the term S_1 vanishes and it is coined by non-uniformity term denoted as S_{non}.

References

1. Lighthill, M. J. On sound generated aerodynamically. I. General theory. *Proc. R. Soc. Lond. Ser. A* **211**, 564-587 (1952).
2. Ostashev, V. E. & Wilson, D. K. *Acoustics in Moving inhomogeneous Medium* (2nd ed.) 25-32 (CRC Press, 2015).
